# Dynamic Serpentine Convolution with Attention Mechanism Enhancement for Beef Cattle Behavior Recognition

**DOI:** 10.3390/ani14030466

**Published:** 2024-01-31

**Authors:** Guangbo Li, Guolong Shi, Changjie Zhu

**Affiliations:** 1 College of Electronic and Information Engineering, Huaibei Institute of Technology, Huaibei 235000, China; 2 School of Information and Artificial Intelligence, Anhui Agricultural University, Hefei 230036, China

**Keywords:** target detection, beef cattle behavior recognition, YOLOv8, dynamic snake-shaped convolution, attention mechanism

## Abstract

**Simple Summary:**

Through beef cattle behavior recognition technology, livestock farmers can promptly identify abnormal behaviors in beef cattle, providing data and technological support for intelligent and welfare-oriented cattle farming. This study is based on computer vision’s convolutional neural networks. It constructs a new method for the real-time detection of beef cattle behavior by optimizing the convolutional module and adding attention mechanisms. The method is evaluated on nine behavior datasets, including standing, lying, mounting, fighting, licking, eating, drinking, walking, and searching. It significantly improves the performance of beef cattle behavior recognition, achieving an average accuracy of 96.5%, which serves as a foundation for beef cattle health assessment and information-based farming.

**Abstract:**

Behavior recognition in beef cattle is a crucial component of beef cattle behavior warning and intelligent farming. Traditional beef cattle behavior recognition faces challenges in both difficulty in identification and low accuracy. In this study, the YOLOv8n_BiF_DSC (Fusion of Dynamic Snake Convolution and BiFormer Attention) algorithm was employed for the non-intrusive recognition of beef cattle behavior. The specific steps are as follows: 45 beef cattle were observed using a fixed camera (A LINE OF DEFENSE) and a mobile phone (Huawei Mate20Pro) to collect and filter posture data, yielding usable videos ranging from 1 to 30 min in length. These videos cover nine different behaviors in various scenarios, including standing, lying, mounting, fighting, licking, eating, drinking, walking, and searching. After data augmentation, the dataset comprised 34,560 samples. The convolutional layer (CONV) was improved by introducing variable convolution and dynamic snake-like convolution modules. The dynamic snake-like convolution, which yielded the best results, expanded the model’s receptive field, dynamically perceived key features of beef cattle behavior, and enhanced the algorithm’s feature extraction capability. Attention mechanism modules, including SE (Squeeze-and-Excitation Networks), CBAM (Convolutional Block Attention Module), CA (Coordinate Attention), and BiFormer (Vision Transformer with Bi-Level Routing Attention), were introduced. The BiFormer attention mechanism, selected for its optimal performance, improved the algorithm’s ability to capture long-distance context dependencies. The model’s computational efficiency was enhanced through dynamic and query-aware perception. Experimental results indicated that YOLOv8n_BiF_DSC achieved the best results among all improved algorithms in terms of accuracy, average precision at IoU 50, and average precision at IoU 50:95. The accuracy of beef cattle behavior recognition reached 93.6%, with the average precision at IoU 50 and IoU 50:95 being 96.5% and 71.5%, respectively. This represents a 5.3%, 5.2%, and 7.1% improvement over the original YOLOv8n. Notably, the average accuracy of recognizing the lying posture of beef cattle reached 98.9%. In conclusion, the YOLOv8n_BiF_DSC algorithm demonstrates excellent performance in feature extraction and high-level data fusion, displaying high robustness and adaptability. It provides theoretical and practical support for the intelligent recognition and management of beef cattle.

## 1. Introduction

With the rapid development of society and the continuous improvement of living standards in the world, urban residents’ demand for beef is on the rise. Simultaneously, there is a growing emphasis on the welfare and production efficiency of beef cattle [1,2]. In beef cattle farming, recognizing cattle behavior in real-time and accurately is essential for sickness-behavior early warning, cattle development assessment, and anomaly monitoring [3,4,5]. The precise identification of behaviors such as standing, lying, mounting, fighting, licking, eating, drinking, walking, and searching provides practitioners with parameters to assess the health status of beef cattle, laying the foundation for modernized beef cattle farming.

Research on beef cattle behavior recognition, both domestically and internationally, falls into two main categories: wearable sensors and non-contact technologies [6]. In wearable sensors recognition, sensors placed on different parts of the cattle collect data on body temperature, sound, pulse, and activity level to infer actual behavior and physiological status [7,8]. While this method reflects cattle behavior to some extent, the use of numerous sensors incurs high material costs, adversely affecting the reliability of data and the accuracy of behavior recognition. Additionally, the critical issue of wearable sensors is limited battery life, which could make no contributions to landfills.

With the emergence of the concept of smart agriculture and the scarcity of agricultural labor, computer vision-based non-contact beef cattle recognition has been continuously evolving [9,10]. This method offers advantages such as non-invasiveness and a high recognition efficiency. The specific contributions of experts, both domestically and internationally, are outlined as follows: Hao et al. [11] proposed an academic approach for individual-cattle recognition based on YOLOv5-EMA. They introduced the Efficient Multi-scale Attention (EMA) module into the backbone of YOLO series detection models to enhance small-object detection performance, achieving an average precision of 95.1%. However, the model’s recognition performance in practical beef cattle farming scenarios with varying levels of occlusion and different lighting conditions needs improvement. Yang et al. [12] introduced deformable convolutions and cooperative attention based on YOLOv8, capturing finer spatial information and essential features of cattle. This method achieved a 72.9% average precision in rapidly detecting cattle behaviors. Zheng et al. [13] proposed an efficient method for dairy cattle behavior recognition, employing channel pruning to reduce model size and parameters. After pruning, the model maintained a high average accuracy of 88.4% for four behaviors in beef cattle: standing, lying, drinking, and walking. However, there is still considerable room for improvement in the recognition accuracy of this model. Wang et al. [14] introduced an efficient 3D-CNN for identifying basic movements in dairy cows, such as lying, standing, walking, drinking, and feeding. The algorithm, based on the SandGlass-3D module with 3D convolution combined with Dwise, efficiently processes spatiotemporal information in videos. ECA attention is also introduced to filter channel information for improved accuracy. However, this model recognizes fewer beef cattle behaviors, limiting its applicability. Shang et al. [15] proposed a lightweight network structure based on feature fusion. This structure enhances the network architecture with SE and CBAM attention mechanisms and achieves feature fusion by combining them. Using a joint loss function, the model achieves a high-precision recognition of beef cattle behaviors such as standing, walking, feeding, and lying. Nevertheless, this model recognizes fewer beef cattle behaviors and operates in a relatively limited environment. Shu et al. [16] introduced a YOLOv5-based model that enhances training efficiency through transfer learning and efficiently recognizes behaviors such as drinking, eating, crawling, and standing in dairy cows. However, this model recognizes fewer beef cattle behaviors and is limited by its simplicity in recognizing environments, making it challenging to meet the requirements of practical beef cattle farming scenarios. Lee et al. [17] proposed a deep learning system for individual cattle recognition based on cattle mouthprint images. By employing transfer learning with different optimizers, the model achieved improved average accuracy in individual-cattle recognition. However, obtaining cattle mouthprint images is challenging, making it difficult to implement in actual beef cattle farming. Tassinari et al. [18] presented a computer vision approach based on deep learning for detecting cows in free-range barns. This method achieved 66% average accuracy in identifying the positions, actions, and movements of cows. There is significant room for improvement in the model’s accuracy.

In summary, researchers have made certain achievements in non-contact beef cattle behavior recognition. However, there is still room for improvement in the following aspects: ① Regarding datasets, existing studies often feature a limited variety of cattle poses, making it challenging to comprehensively reflect the cattle’s growth status. In this study, we established a dataset comprising nine behaviors, including standing, lying, mounting, fighting, licking, eating, drinking, walking, and searching. This dataset provides essential data support for beef cattle behavior recognition and modernized cattle farming. ② In practical beef cattle farming scenarios, the existing beef cattle behavior recognition algorithms have certain limitations. They tend to have higher noise and perform poorly in complex lighting conditions such as strong light, low light, moderate density, and high density. ③ Although previous research studies have achieved a partial recognition of beef cattle behavior in certain scenarios, they have limited recognition of scenes and behaviors, and the algorithm’s accuracy needs further improvement. In this paper, we propose improvements based on the YOLOv8 algorithm to address these limitations. Specifically, we enhanced the model’s feature extraction capability and expanded the receptive field by introducing a dynamic snake-shaped convolution module. Additionally, we incorporated the BiFormer attention mechanism to dynamically learn key features and improve the average accuracy of beef cattle behavior recognition while reducing cases of missed detection, false detection, and low classification confidence.

Based on the aforementioned issues, the research objectives of this study are outlined as follows:

(1) Theoretical Objectives: To establish a more comprehensive dataset for beef cattle posture and enhance the model’s recognition performance by improving the convolution modules and incorporating attention modules on the basis of the YOLOv8 algorithm;
(2) Practical Objectives: To enhance the model’s accuracy in recognizing beef cattle behavior in complex scenarios such as strong light, low light, moderate density, and high density through both dataset augmentation and algorithm optimization. This aims to provide a practical foundation for subsequent applications, including beef cattle disease early warning and intelligent farming.

## 2. Materials and Methods

### 2.1. Dataset

#### 2.1.1. Collection of Dataset

The experimental samples were collected on-site at a beef cattle farm (Shengtu Livestock Farm). The equipment used for beef cattle behavior collection consisted of A LINE OF DEFENSE cameras for fixed recording and a Huawei Mate20 Pro (Huawei Technologies Co., Ltd., Shenzhen, Guangdong, China) mobile phone for free recording. The A LINE OF DEFENSE camera recorded data using a Hikvision Digital Technology Corporation Network Video Recorder (Hangzhou, Zhejiang, China), while data from the Huawei Mate20Pro were stored in the phone’s built-in memory. Both devices can directly export video data.

Communication with the livestock personnel provided essential information about the breeding conditions of the beef cattle:

1. Quantity of Livestock: The maximum number of beef cattle in this enclosure does not exceed 100.
2. Average Activity Area for Beef Cattle: The dimensions of the cattle enclosure allow for a simple calculation indicating that the average activity area per beef cattle is not less than 24.3 square meters.
3. Feeding and Water Access: Beef cattle are fed at around 5:30 a.m. and 6:00 p.m., with two feedings per day. Water for the cattle is freely accessible in the designated water area.
4. Ventilation Conditions and Livestock Environment: Natural ventilation is employed, and the environment for beef cattle rearing is in its original state without any special padding.

In order to acquire more effective data on beef cattle behavior, this study captured videos of daily cattle behavior in different scenarios over a span of 7 days (11–17 November 2023). These videos aimed to comprehensively reflect various characteristics of beef cattle behavior. Simultaneously, manual annotations were applied to segments of videos depicting nine behaviors of beef cattle: standing, lying, mounting, fighting, licking, eating, drinking, walking, and searching. The video durations varied from 1 to 30 min. The recognition rules and examples for the nine beef-cattle behaviors are presented in Table 1.

**Figure 1:**
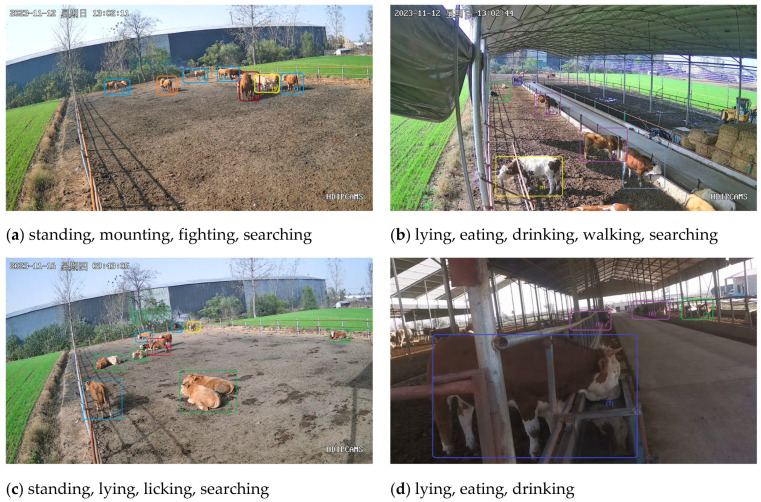
Schematic of beef cow behavior (“星期日” is Sunday. “星期四” is Thursday. Same color for same beef cattle behavior).

As depicted in Figure 1, the annotated beef cattle targets labeled (1) through (9) correspond to standing, lying, mounting, fighting, licking, eating, drinking, walking, and searching behaviors of beef cattle, respectively.

The beef-cattle-behavior data collection process is illustrated in the following flowchart.

**Figure 2:**
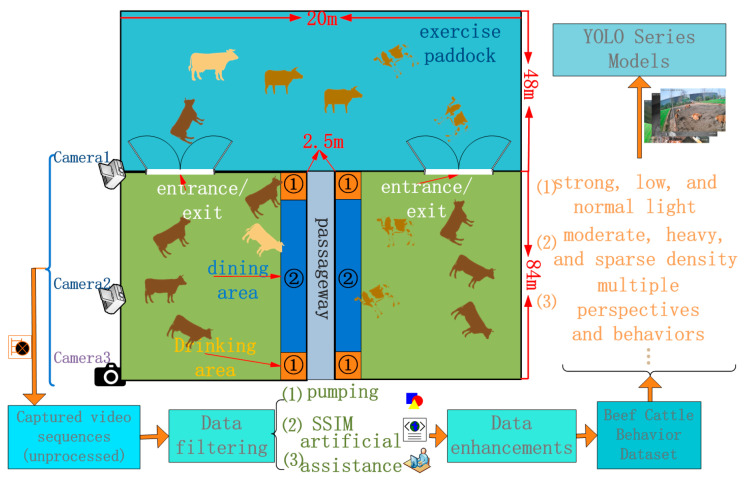
Acquisition flowchart.

As indicated in Figure 2, the beef cattle facility primarily comprises an exercise paddock, entrances and exits, a dining area, and a drinking area, denoted as (1) for the drinking area and (2) for the dining area. The process of beef cattle behavior collection and processing is outlined as follows: (1) initial video sequences of beef cattle are captured with cameras. Cameras 1 and 2 are fixed at designated positions (Figure 1a–c), capturing overall cattle behavior. Camera 3 is manually positioned (Figure 1d), capturing localized features of beef cattle behavior. (2) Data filtering involves preprocessing the collected video sequences through three main steps: firstly, frame subsampling to reduce the similarity between adjacent frames; secondly, enhancing data diversity through Structural Similarity (SSIM) [19] technology; and finally, a manual auxiliary filtering process. Details are provided in the subsequent Section 2.1.2. (3) Data augmentation: following data filtering, the selected data undergo augmentation to expand the dataset. Refer to the subsequent section, Section 2.1.2, for specific details. (4) Input to the YOLOv8n Series Improvement Algorithm: the beef cattle behavior dataset, encompassing different lighting conditions (strong light, low light, and normal light), varied density levels (moderate, heavy, and sparse), and multiple angles of cattle behavior, is input into the YOLOv8n series improvement algorithm.

#### 2.1.2. Data Preprocessing and Enhancement

Due to the low activity levels of beef cattle behaviors such as standing, lying, and searching, with minimal differentiation between adjacent temporal samples, an image is extracted every 20 frames. Simultaneously, the Structural Similarity Index (SSIM) is employed to filter sample images.

SSIM (Structural Similarity Index) is a metric for assessing image quality, enabling the evaluation of the similarity between two images. The assessment criteria primarily focus on image luminance, contrast, and structure. Luminance and contrast in the images are determined by the mean grayscale and standard deviation of grayscale, respectively.

**Figure 3:**
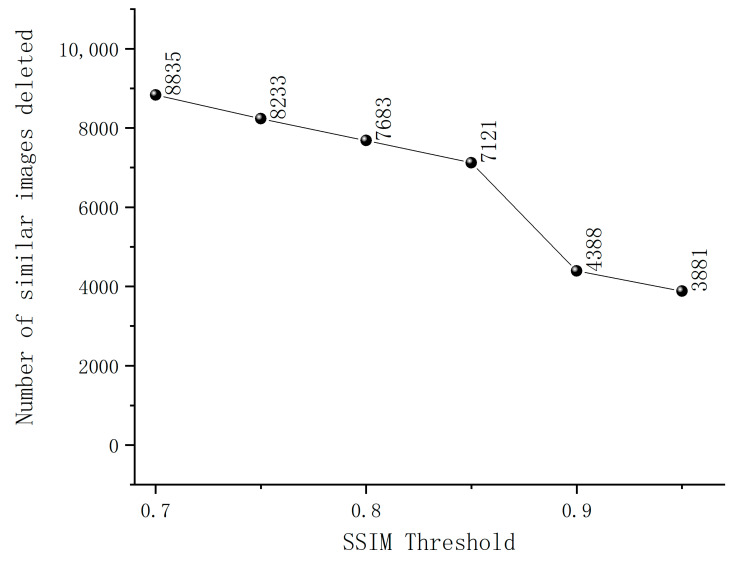
The quantity of similar images removed at different SSIM thresholds.

The criterion for selecting sample images using SSIM is that a higher SSIM value indicates smaller differences between the two images and a higher image similarity. After data frame extraction, the dataset comprised 12,215 frames. As shown in Figure 3, the SSIM curve remains relatively stable within the SSIM threshold ranges of 0.7–0.85 and 0.9–0.95. However, within the SSIM threshold range of 0.85–0.9, the curve exhibits a noticeable decline. This suggests that images within this threshold range exhibit significant similarity, and at an SSIM threshold of 0.85, the maximum number of similar images is eliminated. Therefore, this study set the SSIM threshold at 0.85. If the SSIM value between two sample images is greater than 0.85, it indicates a high level of similarity, and only one of the sample images is retained; otherwise, all images are preserved.

To ensure that the dataset aligns with actual beef cattle farming practices, communication with livestock keepers revealed that maintaining multiple cattle in a single enclosure is more economically beneficial for the farm. Therefore, this study primarily collected samples with a high level of density. Simultaneously, to enhance the robustness of the dataset, a small number of samples with a low level of density were also collected. The beef cattle behaviors were annotated using a labeling software.

**Figure 4:**
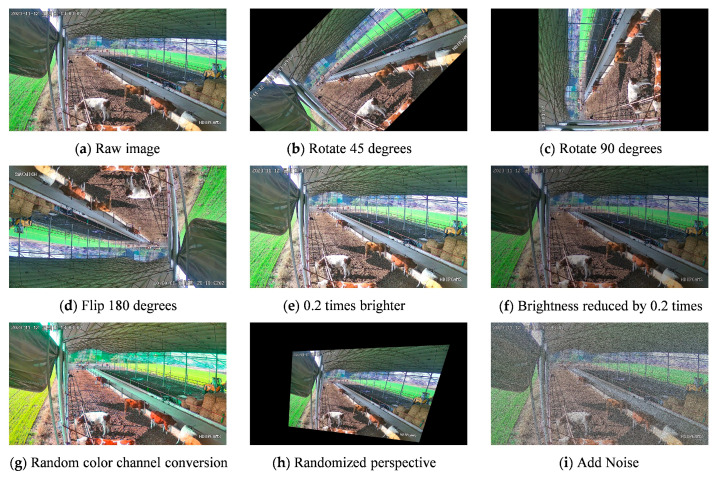
Data enhancement example (“星期日” is Sunday).

The collected data were filtered and integrated, resulting in a total of 4320 frames of valid images representing nine categories of beef cattle postures. Subsequently, the original dataset underwent various data augmentations, including clockwise rotations of 45 and 90 degrees, a 180-degree flip, brightness adjustments of +0.2 and −0.2, random color channel transformations, random perspective changes, and the addition of noise. This data augmentation process expanded the experimental samples to eight times their original size, as illustrated in Figure 4. The applied data augmentations enhanced the algorithm’s ability for object detection in complex scenes, providing more information on beef cattle posture characteristics. For instance, random color channel transformation can simulate changes in lighting conditions, introducing image noise and mitigating the impact of lighting factors to enhance algorithm robustness. Finally, the experimental samples were divided into training and testing sets in an 8:2 ratio.

**Figure 5:**
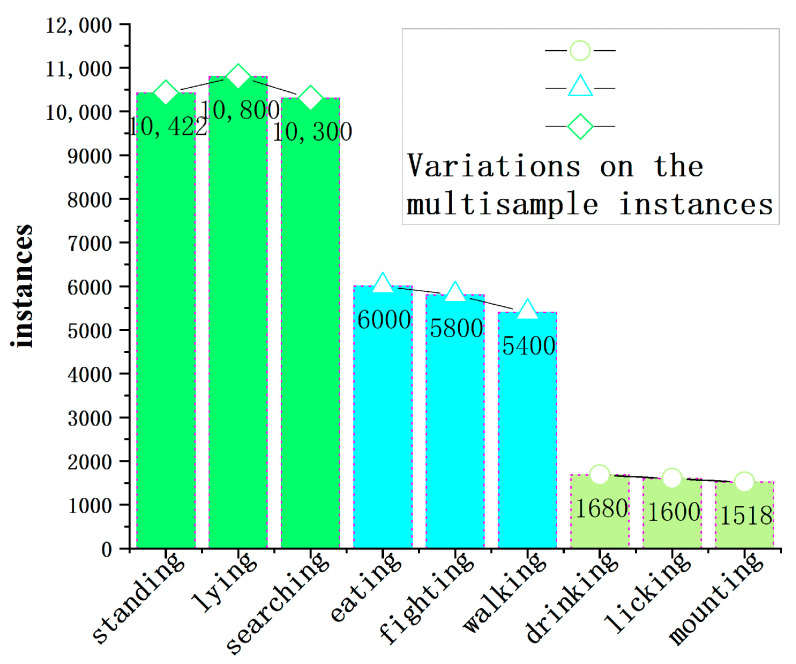
Distribution of instances of beef cattle behavioral categories.

In the training of a model on a dataset, the quantity of instances for each category plays a crucial role in the algorithm’s training. Figure 5 illustrates the distribution of instance quantities for each category in the training set. From the bar chart, it can be observed that the instances of beef cattle standing, lying, and engaging in searching behavior are approximately 10,500, while instances of eating, fighting, and walking behaviors are around 5800. Instances of drinking, licking, and mounting behaviors are approximately 1600. The line chart reveals that the instances for standing, lying, and searching behaviors, as well as eating, fighting, and walking behaviors, and drinking, licking, and mounting behaviors, remain stable. The frequency of the nine cattle behaviors generally aligns with the daily farming activities of beef cattle, indicating a reasonable distribution of samples in the dataset. This distribution provides valuable data support for subsequent algorithm models to explore features of beef cattle behavior.

### 2.2. Principle of the YOLOv8 Algorithm

The YOLOv8 is the latest SOTA single-stage object detection algorithm, integrating and optimizing the essence of the YOLO series algorithms [20,21,22,23]. YOLOv8 innovates in the backbone network, detection head, loss function, and other aspects, resulting in varying degrees of improvement in detection accuracy compared to YOLOv5, YOLOv6, and YOLOv7 [24]. Simultaneously, YOLOv8 provides a unified model training framework, greatly enhancing the algorithm’s scalability. To meet different scene requirements, YOLOv8 offers five model versions: YOLOv8n, YOLOv8s, YOLOv8m, YOLOv8l, and YOLOv8x. These models increase in depth and width based on YOLOv8n. Among them, the YOLOv8n model is the smallest and has the fastest detection speed, while the YOLOv8x model is the largest, with the highest detection accuracy but the slowest speed. Considering the application of beef cattle posture recognition in practical projects, the YOLOv8n model, which is the smallest, was chosen. Its structure mainly consists of four parts: the input network, the backbone network, the neck network, and the output network.

**Figure 6:**
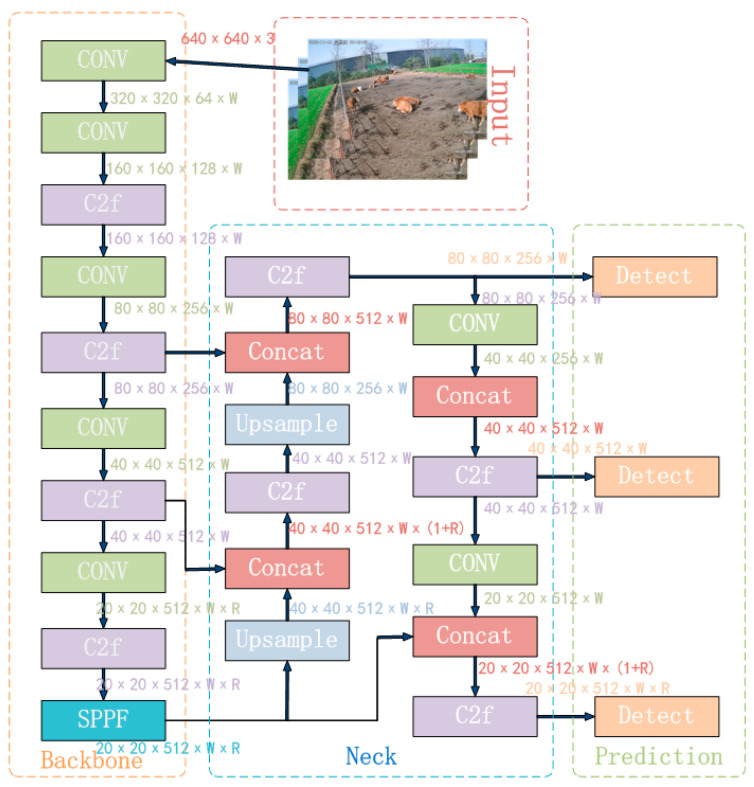
YOLOv8n algorithm flowchart.

The input network primarily processes input images to a uniform resolution. The backbone network extracts features through a convolutional neural network. In YOLOv8n, the C3 structure is replaced with the C2f structure to enrich the gradient flow, and the channel number is optimized to better adapt to multi-scale models. The neck network still adopts the commonly used PAN-FPN, which preserves information from different feature levels. However, by introducing attention modules, the global and local feature correlations are further enhanced, facilitating more efficient information fusion and strengthening the propagation of information across high and low levels. The output network mainly outputs the position and category features of the target through classification and regression. The complete YOLOv8n algorithm model is illustrated in Figure 6.

### 2.3. The Improvement of YOLO Algorithm

#### 2.3.1. Introducing the Dynamic Serpentine Convolution DSConv Module

Due to the finite composition of pixel elements in the input images, the structural components of beef cattle behavior occupy only a small portion of the entire image. Moreover, these behavioral structures are susceptible to interference from complex backgrounds in the farming environment. Consequently, the model faces challenges in extracting subtle feature variations, leading to a suboptimal detection performance and low recognition efficiency. Simultaneously, the posture structure of beef cattle is intricate and variable. The same posture may exhibit different feature expressions in diverse scenarios, such as varying angles and illumination conditions. Therefore, the fundamental aspect of beef cattle posture recognition lies in the extraction of key features.

Inspired by Deformable Convolution [25], the model adapts convolutional kernels’ shapes during feature learning to focus on the essential structural features of beef cattle posture. However, given the small proportion of core structural features in the posture and the risk of convolutional kernels drifting away from the posture structure, the model inevitably loses posture structural awareness. To address this, we introduce the Dynamic Snake Convolution module [26], which efficiently extracts key features while adhering to the target structure under constrained conditions in beef cattle posture recognition.

Dynamic Snake Convolution (DSConv) extracts local features in beef cattle posture through the following process: for a standard 2D convolution with coordinates *K*, where the central coordinate is *K_i_* = (*x_i_*, *y_i_*) (*i* is an integer), and a dilation rate of 1, the 3 × 3 convolution kernel is represented as:

(1) K={(x−1,y−1),(x,y−1),⋯,(x+1,y+1)}

**Figure 7:**
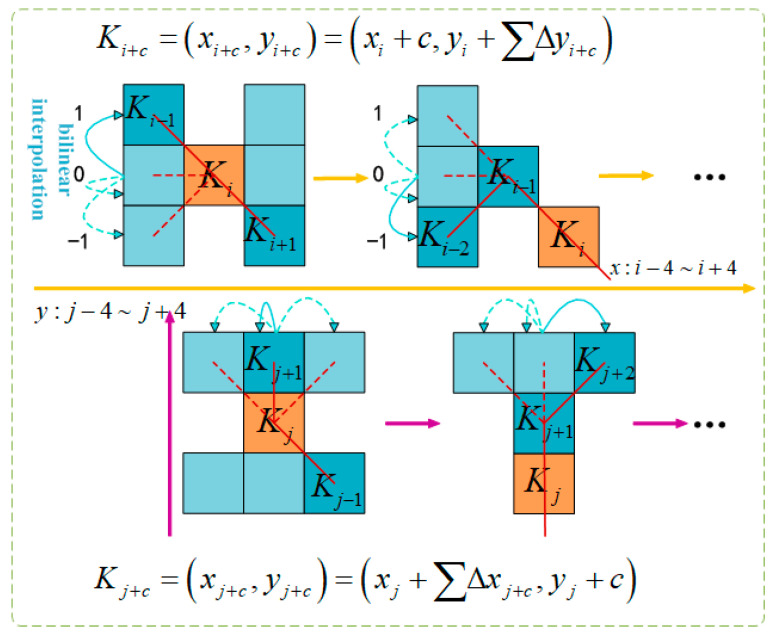
Graphical representation of DSConv coordinate calculation.

To make the convolutional kernel more adaptable to the complex geometric features of beef cattle posture, a deformation offset ∆ is introduced. However, if the model learns deformation offsets randomly, the perceived area may deviate from the target, especially in complex beef cattle postures. Therefore, this paper adopts the iterative strategy shown in Figure 7. This strategy sequentially matches each target to observable positions, ensuring continuous feature attention without excessively spreading the perception area due to large deformation offsets.

In DSConv, straighten the standard convolutional kernel in the *x*- and *y*-axes directions. Using a 9-size convolutional kernel, for the *x*-axis, each specific position *K_i_* ± *c* is calculated as: (*x_i_* ± *c*, *y_i_* ± *c*), where *c* = {0, 1, 2, 3, 4} denotes the horizontal distance from the central grid. The selection of each grid position *K_i_* ± *c* in the convolutional kernel *K* is an accumulative process. Starting from the central position *K_i_*, each subsequent position *K_i_* + 1 depends on the previous grid position and increases by an offset Δ = {*δ*|*δ*ε[−1, 1]}. This cumulative offset Σ ensures the convolutional kernel aligns with the morphological structure of beef cattle posture features in the *x*-axis direction.

(2) $$K_{i\pm c}=\left\{\begin{array}{l} \left(x_{i+c},y_{i+c}\right)=\left(x_{i}+c,y_{i}+\Sigma_{i}^{i+c}\Delta y\right)\\ \left(x_{i-c},y_{i-c}\right)=\left(x_{i}-c,y_{i}+\Sigma_{i-c}^{i}\Delta y\right) \end{array}\right.$$

Equation (2) is modified in the *y*-axis direction as follows:

(3) $$K_{j\pm c}=\left\{\begin{array}{l} \left(x_{j+c},y_{j+c}\right)=\left(x_{j}+\Sigma_{j}^{j+c}\Delta x,y_{j}+c\right)\\ \left(x_{j-c},y_{j-c}\right)=\left(x_{j}+\Sigma_{j-c}^{j}\Delta x,y_{j}-c\right) \end{array}\right.$$

Due to the fact that the offset Δ is typically a decimal, the implementation of a bilinear interpolation is as follows:

(4) $$K=\Sigma_{K^{\prime }}B\left(K^{\prime },K\right)\cdot K^{\prime }$$

Here, *K* represents the fractional positions in Equations (2) and (3), and *K*′ enumerates all integral spatial positions. *B* is the bilinear interpolation kernel, which is decomposed into two one-dimensional kernels, as follows:

(5) $$B\left(K,K^{\prime }\right)=b\left(K_{x},K_{x}^{\prime }\right)\cdot b\left(K_{y},K_{y}^{\prime }\right)$$

**Figure 8:**
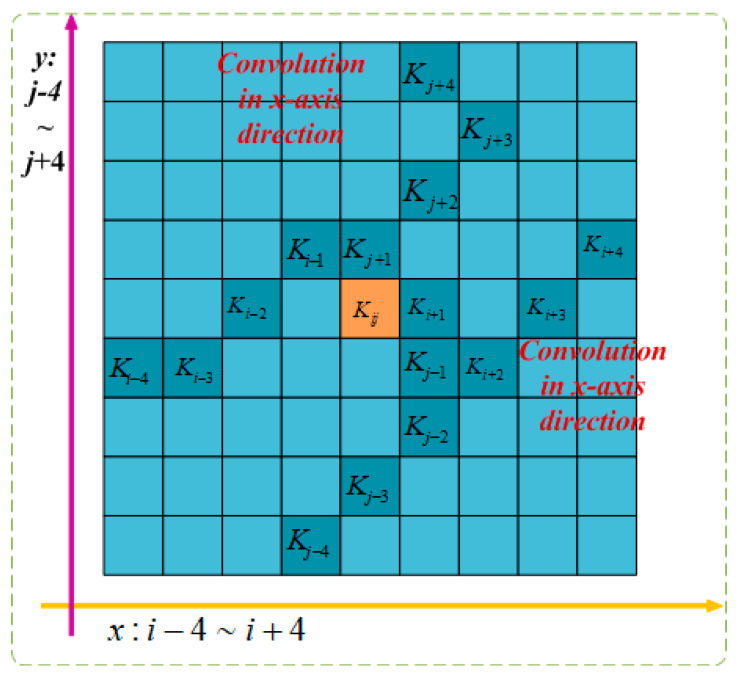
DSConv feeling wild.

As depicted in Figure 8, due to the two-dimensional (*x*-axis, *y*-axis) variations, DSConv covers a range of 9 × 9 during the deformation process, expanding the model’s receptive field. This adaptation to dynamic structure better suits the morphological aspects of beef cattle posture, enhancing the perception of key features and laying the foundation for accurate posture identification.

#### 2.3.2. Introducing the Dynamic Serpentine Convolution DSConv Module

To better capture feature information in images, this study introduced an attention mechanism. This mechanism effectively captures global and local connections and calculates and emphasizes essential image features, reducing feature loss in scenarios with small targets or severe occlusion, thereby improving network performance. However, introducing attention mechanisms in networks can lead to issues such as a high memory consumption and computational overhead. Therefore, this study introduced a lightweight and efficient BiFormer [27] attention mechanism to address these concerns.

BiFormer possesses dynamic sparse characteristics and combines with an efficient pyramid network. It queries to perceive and filter out weakly correlated tokens, allocating efficient computation. Even in challenging scenarios such as small targets and severe occlusion, BiFormer maintains outstanding performance.

**Figure 9:**
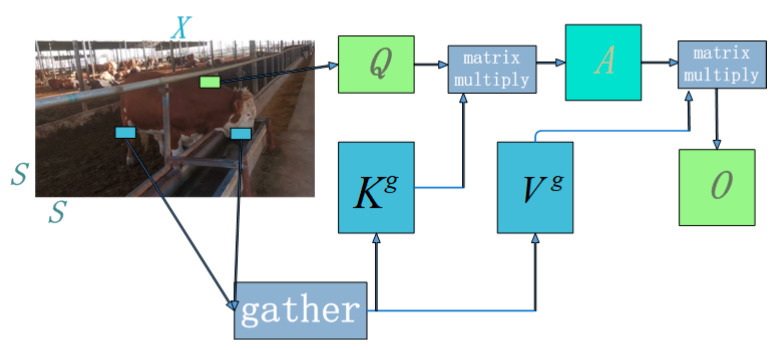
Bilayer routing attention mechanism.

The dynamic sparse characteristics of BiFormer are achieved through the implementation of a two-layer Routing Attention (BRA). It filters out unrelated key–value pairs in coarse-grained regions, retains a small number of candidate routing areas, and applies attention mechanisms at the token level to obtain pixel-level focused regions. The specific process of the two-layer Routing Attention is illustrated in Figure 9. The input two-dimensional feature map *X* is divided into non-overlapping regions of size *S* × *S*, and tensors *Q*, *K*, and *V* are obtained through a linear projection as per Equations (6)–(8). The projection weights for each tensor are represented by $W^{q}$, $W^{k}$, and $W^{v}$, where the non-overlapping regions in the input feature map are denoted by $X^{r}$.

(6) $$Q=X^{r}W^{q}$$

(7) $$K=X^{r}W^{k}$$

(8) $$V=X^{r}W^{v}$$

To precisely focus on the non-overlapping regions of the input feature map, we compute the mean of *Q* and *K* for the non-overlapping regions to obtain the query and key vectors *Q^r^*, *K^r^*. Multiplying the key vectors *Q^r^* and ${\left(K^{r}\right)}^{T}$ yields the correlation matrix *A^r^* between non-overlapping regions (Equation (9)), in which *T* represents the transpose. We then apply the *topK* algorithm to filter out weak correlations, keeping only the *topK* strong connections. This process generates the routing index matrix *I^r^* (Equation (10)).

(9) $$A^{r}=Q^{r}{\left(K^{r}\right)}^{T}$$

(10) $$I^{r}=topK\left(A^{r}\right)$$

To efficiently handle non-overlapping regions dispersed across the feature map, the key-value pair tensors *K* and *V* are collected. Data is extracted and new data tensors *K^g^* (as per Equation (11)) and *V^g^* (as per Equation (12)) are generated using the *gather* ( , ) function. The final output feature map, denoted as *O*, is obtained through the attention mechanism.

(11) $$K^{g}=gather\left(K,I^{r}\right)$$

(12) $$V^{g}=gather\left(V,I^{r}\right)$$

(13) $$O=Attention\left(Q,K^{g},V^{g}\right)$$

**Figure 10:**
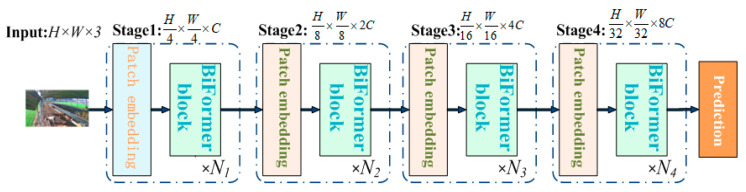
BiFormer overall structure.

Based on the BRA module, a novel universal vision transformer called BiFormer is constructed using the four-level pyramid structure of a vision transformer [28], as illustrated in Figure 10.

Initially, in the first stage, overlapping blocks are embedded, and in the second to fourth stages, the input space resolution is optimized through block merging modules. This process doubles the channel count and undergoes feature transformation through *Ni* consecutive BiFormer blocks.

**Figure 11:**
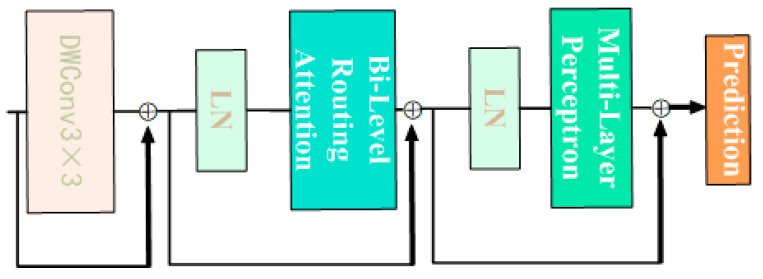
Detailed structure of BiFormer module.

**Figure 12:**
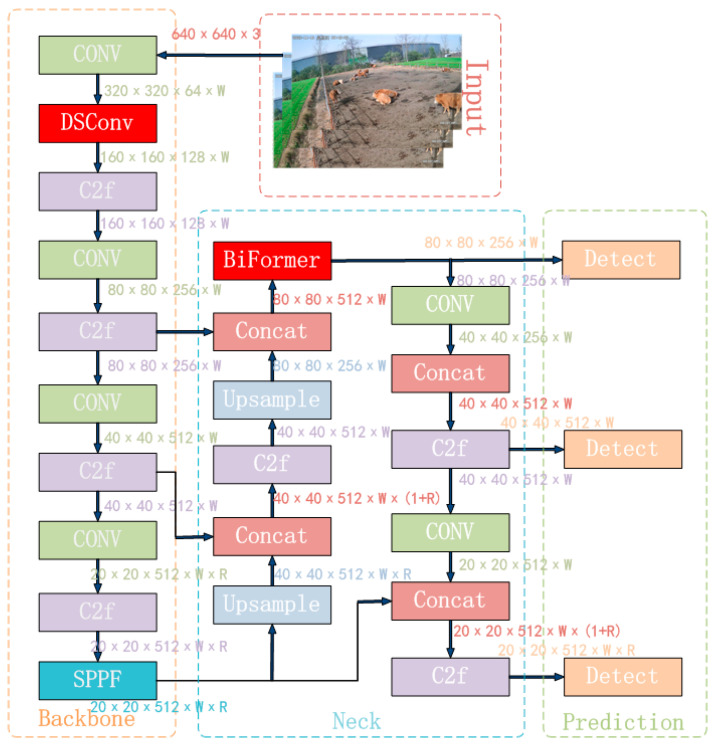
YOLOv8n_BiF_DSC algorithm flow.

The BiFormer block begins with the implicit encoding of relative positional information using a 3 × 3 depthwise convolution. Subsequently, a two-layer Routing Attention (BRA) module is applied for modeling cross-positional relationships, and full position embeddings are achieved through a multi-layer perceptron (MLP) module, as illustrated in Figure 11. The modified YOLOv8n_BiF_DSC model, incorporating improvements from DSConv and BiFormer, is depicted in Figure 12.

## 3. Results and Analysis

### 3.1. Experimental Environment

The high number of model parameters and computational complexity in computer vision detection algorithm networks can significantly affect the training speed of the network. Therefore, this study employed GPUs with high data throughputs to greatly accelerate network training [29]. The experiments reported in this paper used the NVIDIA GeForce RTX 4070 graphics card with 16 GB of video memory and an Intel Core i7-13700F 3.0 GHz (Intel Corporation, Santa Clara, CA, USA) processor with 16 GB of memory. Experiments were conducted in CUDA 11.8, Cudnn8700, and PyTorch 2.0.0 environments. Specific hardware and software configuration details are outlined in Table 2 below.

To train the algorithm, we used the large COCO dataset for pre-training, resulting in the weight file [30]. Our model network parameters were optimized using the SGD optimizer as a whole. The training batch was set at 32, the initial learning rate was set at 0.002, the momentum was set at 0.94, and the weight decay coefficient was set at 0.0015. In addition, the cosine annealing algorithm was used to adjust the learning rate. The algorithm iterated 200 times, and the image input size was 640 × 640 pixels.

### 3.2. Model Evaluation

This study used a series of common evaluation indicators to evaluate object detection algorithms. Specifically, *P* represents the proportion of positive samples, or the accuracy rate; *R* represents the proportion of positive samples, or the recall rate; *AP* represents the average accuracy; mAP refers to the average accuracy across all categories; and IOU stands for Intersection over Union threshold, which can be set to values between 0.5 and 0.95, with corresponding mAP50 and mAP50:95 values. The model’s performance is further assessed by the following metrics: *TP*, number of correct target detections; *FP*, number of false alarms; *FN*, number of missed alarms; and mean accuracy (mAP).

(14) $$Precision=\frac{TP}{TP+FP}$$

(15) $$Recall=\frac{TP}{TP+FN}$$

(16) $$AP=\int_{0}^{1}P(R)dR$$

### 3.3. Introducing Dynamic Serpentine Convolution Recognition Results

In convolutional neural networks, the feature extraction effect of convolutional layers directly influences the model recognition performance and accuracy. Regular convolution can extract features, but the extracted feature areas are scattered. Deformable convolution provides the convolution kernel with deformation characteristics, automatically adjusts the shape to better adapt to the key areas, and extracts the main feature information. This paper presents a dynamic serpentine convolution (DSConv) that is more suitable for extracting cattle pose characteristics and compares it with regular convolution and deformable convolution (DConv). The comparison results are shown in Table 3 below.

In terms of improving the performance of the algorithm, the incorporation of the deformable convolution YOLOv8n_DC and dynamic serpentine convolution YOLOv8n_DSC, as compared to regular convolution YOLOv8n, has distinct degrees of improvement in accuracy, recall rate, and mean accuracy. This indicates that deformable convolution, dynamic serpentine convolution, and cattle pose feature extraction are positively correlated. To further improve the algorithm’s performance, the recall rate of YOLOv8n_DSC closely resembles the improvement rate of YOLOv8n_DC. In addition, the 50:95 YOLOv8n_DSC accuracy and mean accuracy increased by 5%, 3.2%, and 3.3%, respectively, compared to the original version. Therefore, this study chose the YOLOv8n_DSC model, which incorporates dynamic serpentine convolution, to enhance the model’s focus on key feature areas of the input image, ensure feature continuity through dynamic serpentine convolution kernels, and expand the model’s receptive field. This strategy provides a solid database to improve the performance of cattle pose detection.

### 3.4. Introducing Dynamic Serpentine Convolution Recognition Results

Well-designed attention mechanisms can greatly improve neural networks’ ability to handle sequential and image data, accurately process information, and boost overall model performance. This study introduces a lightweight and efficient BiFormer attention mechanism for these purposes. It also provides a comparison with several common attention mechanisms, including SE [31], CBAM [32], and CA [33], as illustrated in Table 4.

When comparing the YOLOv8n_SE model’s accuracy, recall rate, and average accuracy rate with its original version, it is clear that while none of these metrics significantly improved, there was a slight decrease. The reason for this is that the SE attention mechanism only considers parameter information in the channel dimension but ignores parameter information in the spatial dimension. This lack of emphasis on spatial information results in subpar recognition performance. In contrast, the YOLOv8n_CBAM, YOLOv8n_CA, and YOLOv8n_BiF models, each with their unique attention mechanisms, showed significant improvements in accuracy, recall rate, and average accuracy compared to the original version. Interestingly, the YOLOv8n_BiF model had the most significant improvement in recall rate and average accuracy rate for the beef cattle dataset. For the 50:95 and 50:50 data splits, the recall rate and average accuracy rate improved by 6.5% and 2.7%, respectively, indicating that the BiFormer attention mechanism is particularly sensitive to cattle behavior and is better suited to the model for extracting feature information that increases recognition accuracy.

### 3.5. Results and Analysis of Ablation Experiment

The results of the experiment to assess the effect of using different upgraded modules simultaneously on the recognition of the posture of beef cattle are presented in Table 5. The “-” sign indicates the absence of a particular module implementation, while the “√” sign indicates its inclusion. This experiment used the control variable method, ensuring that the only difference between the experimental and control groups was the module(s) being tested. The results indicate that the introduction of the dynamic serpentine convolution (DSConv) and BiFormer attention mechanism resulted in notable improvements in accuracy, recall rate, and mean accuracy of 50 and 50:90 when compared to the original YOLOv8n. This proves the effectiveness of dynamic serpentine convolution in enhancing the model’s extraction of beef cattle posture characteristics, feature utilization rate, model receptive field, and posture recognition prediction effect. In addition, the BiFormer attention mechanism offers dynamic and sparse features, improving the model’s recognition effectiveness and enhancing its robustness against challenging scenarios such as small targets and severe occlusion.

In terms of algorithm optimization, the YOLOv8n_BiF_DSC algorithm, which incorporates two improvement points, exhibits significantly higher accuracy and average accuracy at thresholds of 50, 50:95 compared to YOLOv8n_DSC and YOLOv8n_BiF, which only incorporate one improvement point. The improvement in average accuracy at the 50:95 thresholds is notably higher than the average accuracy at 50, with a magnitude of 7.1%. This indicates that YOLOv8n_BiF_DSC performs well in recognition when the IOU threshold is relatively large, demonstrating high robustness. Regarding recall rates, YOLOv8n_BiF_DSC is slightly lower than YOLOv8n_BiF but still superior to the original YOLOv8n and YOLOv8n_DSC versions. Overall, despite having a suboptimal recall rate, the YOLOv8n_BiF_DSC algorithm achieves optimal values in terms of accuracy and average accuracy at thresholds of 50, 50:95, reaching 93.6%, 96.5%, and 71.5%, respectively. This represents an improvement of 5.3%, 5.2%, and 7.1% over the original YOLOv8n, further highlighting the feasibility of the YOLOv8n_BiF_DSC algorithm in recognizing beef cattle postures.

**Figure 13:**
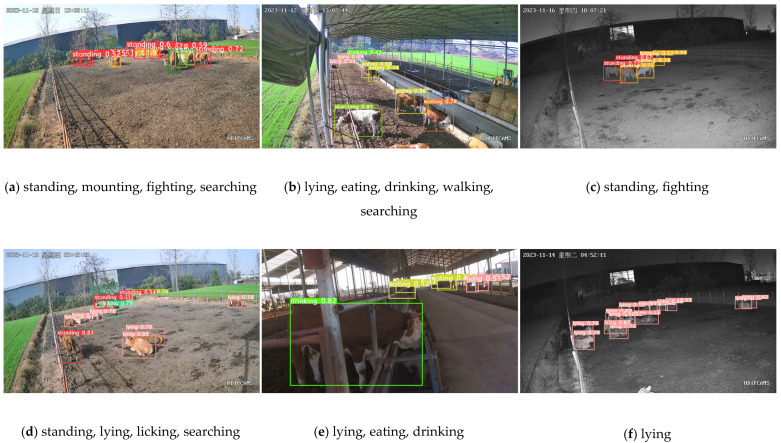
Loss curve.

In Figure 13, the performance variation of each algorithm in ablation experiments on the test set is shown. The horizontal axis represents the number of iterations, while the vertical axis denotes the magnitude of the loss. Initially, the algorithm’s loss escalates rapidly but later fluctuates, eventually reaching equilibrium at about 0.025–0.03. The training process showed no signs of underfitting or overfitting.

### 3.6. Comparative Analysis with Other Models

To validate the effectiveness of the YOLOv8n_BiF_DSC algorithm, experiments were conducted using FastRCN [34], YOLOX [35], and YOLOv7 methods. Consistent experimental metrics, datasets, and conditions were used, and variations are presented in Table 6. The results show that the proposed YOLOv8n_BiF_DSC protocol outperforms FastRCN, YOLOX, and YOLOv7 in the postural recognition of beef cattle, with YOLOv7 having a significantly increased performance over FastRCN and YOLOX. The enhanced algorithm conveys a superior accuracy, recall rate, and average performance over YOLOv7, particularly at the 50:95 threshold, resulting in a notable 6.7% and 1% increase in postural recognition of beef cattle, respectively. These findings confirm the promising potential of the YOLOv8n_BiF_DSC algorithm incorporating DSConv and BiFormer attention mechanisms for the analysis of cattle behavior.

### 3.7. YOLOv8n_BiF_DSC Test Results

**Figure 14:**
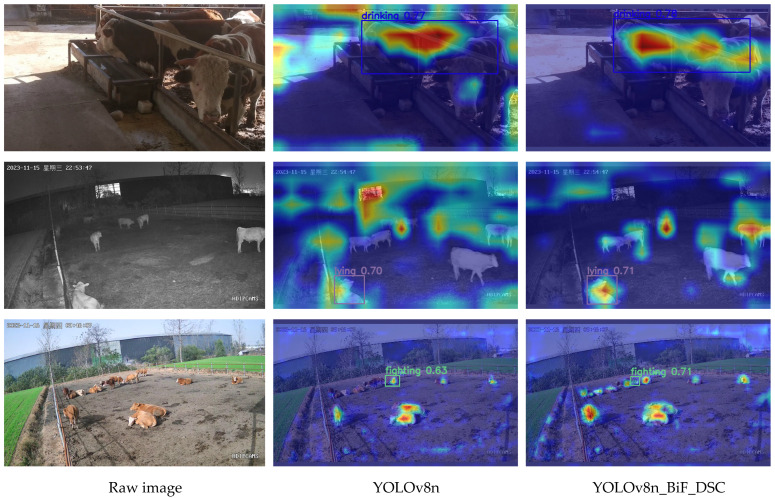
YOLOv8n_BiF_DSC beef cattle behavioral detection chart.

Figure 14 shows the effect of YOLOv8n_BiF_DSC 9 on the postural recognition of beef cattle.

As can be seen from the graphs above, Figure 14a,b,d display recognition results at different points in time from a fixed position. Despite the fact that some beef cattle postures at a substantial distance in Figure 14b went unnoticed, the algorithm did manage to identify standing, lying, mounting, fighting, licking, eating, drinking, walking, and searching behaviors in other images. Figure 14e shows the recognition results of a moving capture, where distant cattle positions are not identified due to feature loss. However, postures such as lying, eating, and drinking are successfully identified. Figure 14c,f show recognition results at night from a fixed position, where some beef cattle positions are partially obscured. Despite this, the YOLOv8n_BiF_DSC algorithm can still accurately detect standing, fighting, and lying behaviors in both images. In conclusion, the YOLOv8n_BiF_DSC algorithm continues to show its ability to accurately recognize multiple beef cattle behaviors across various scenes in practical cattle farming.

### 3.8. Model Visualization Analysis

**Figure 15:**
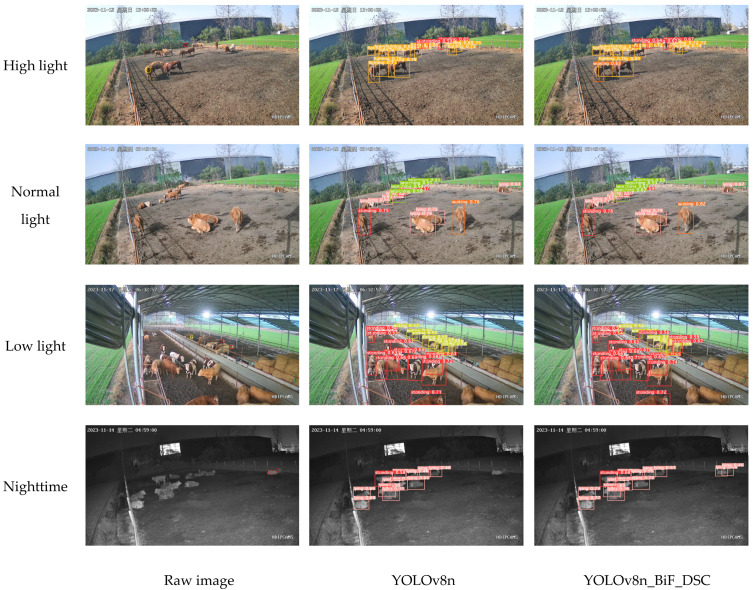
Visualization map.

To better explain the predictive performance of the model, this paper introduces Grad-CAM++ [36] to provide more intuitive visual explanations for the model, as illustrated in Figure 15. The model’s attention to the target regions corresponds to the colors in the image, where blue and red represent indifference and close attention, respectively.

As seen in Figure 15, the original YOLOv8n model tends to overly focus on background noise during the recognition process, to some extent diminishing the model’s recognition accuracy. In contrast, the YOLOv8n_BiF_DSC model utilized in this paper pays more attention to beef cattle behaviors. Despite being affected by some background noise, the features of beef cattle behaviors in the images are successfully extracted. This results in a significant improvement over the original YOLOv8n model, demonstrating the feasibility of accurate beef cattle behavior recognition in practical cattle farming using YOLOv8n_BiF_DSC.

**Figure 16:**
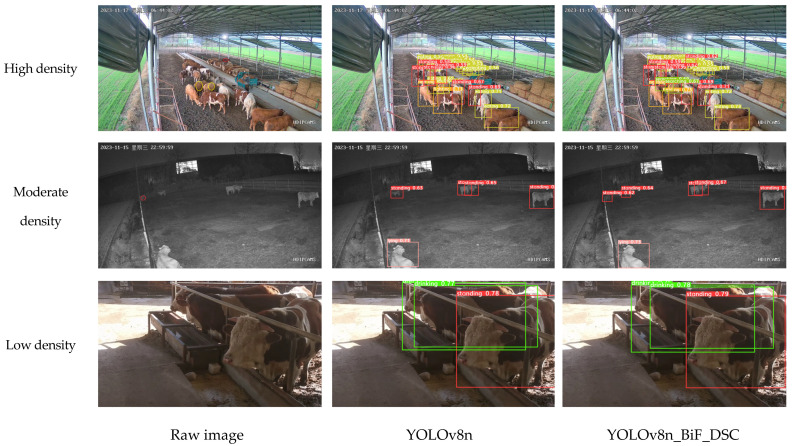
Average accuracy of beef cattle postures in the YOLOv8n_BiF_DSC algorithm.

As depicted in Figure 16, the distribution of average accuracy in beef cattle posture for the YOLOv8n_BiF_DSC algorithm is evident. The average accuracy in recognizing beef cattle lying, mounting, fighting, drinking, licking, standing, eating, walking, and searching behaviors decreases sequentially. Beef cattle lying, mounting, and fighting behaviors exhibit a higher average accuracy in recognition, given their distinctiveness from other behaviors in actual farming practices. Among them, the average accuracy in recognizing the lying behavior is the highest, reaching 0.989. However, distinguishing between beef cattle’s standing, walking, and searching behaviors poses challenges due to shared characteristics. In conditions of substantial occlusion noises in actual farming, misclassifications may occur more frequently, resulting in a slightly lower recognition performance. Nevertheless, even the lowest average accuracy in recognizing the searching behavior reaches 0.93, providing potential for subsequent beef-cattle behavior-warning applications.

### 3.9. Comparison of Recognition Effects of Different Scene Models

#### 3.9.1. Different Lighting Environments

**Figure 17:**
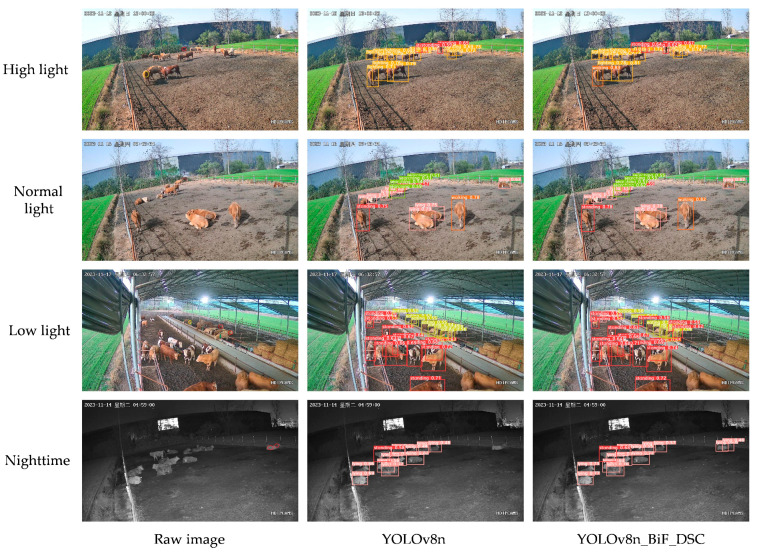
Behavioral detection charts for beef cattle with different light levels.

**Figure 18:**
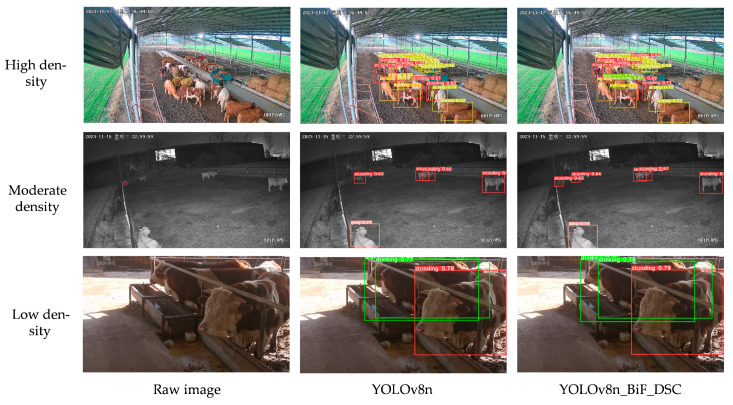
Intensity level charts of beef cattle behavioral detection.

Specific features of beef cattle behavior images are shown to vary significantly under different lighting conditions. Notably, there is an increased presence of noise in both high and low lights compared to normal light. This phenomenon of lighting variations significantly affects the overall recognition performance of the model. In order to effectively evaluate the robustness of our proposed YOLOv8n_BiF_DSC model in beef cattle behavior recognition, we made an in-depth comparison of its performance under different lighting conditions. This comparison involved four conditions: high light, low light, normal light, and nocturnal, with the last two conditions corresponding to evening and nighttime. We compared the results with the original YOLOv8n model. Specific times for different lighting conditions are as follows: high-light conditions typically occur around 11 a.m. to 2 p.m. on a sunny day; low-light conditions around 5.30 p.m. or 6.30 p.m.; normal-light conditions around 9.30 p.m. or 3.30 p.m.; and nighttime corresponds to other dark hours. (In Figure 17 and Figure 18, the red and yellow circles in the original images represent missed detections and false positives, respectively).

From Figure 17, under normal light feeding conditions, both the YOLOv8n_BiF_DSC and YOLOv8n models accurately recognize beef cattle behaviors such as standing, lying, searching, and walking. However, the YOLOv8n_BiF_DSC model exhibits higher confidence in detected classifications, with a more pronounced fitting effect of bounding boxes. Under high-light feeding conditions, where there is an increased presence of image feature noise, YOLOv8n misses one instance of standing behavior and falsely detects one instance of searching behavior compared to the YOLOv8n_BiF_DSC model. In low-light feeding conditions, where an image feature loss is more prevalent, YOLOv8n misses two instances of standing behavior and falsely detects one instance of eating behavior compared to the YOLOv8n_BiF_DSC model. Under nocturnal feeding conditions, YOLOv8n misses two instances of lying behavior compared to the YOLOv8n_BiF_DSC model. In summary, the YOLOv8n_BiF_DSC model maintains robustness and precision in complex lighting environments, scenarios with fewer image feature points, and situations with increased noise.

#### 3.9.2. Different Intensities

Different levels of beef cattle density result in varying degrees of cattle occlusion and significant differences in background interference factors. Generally, the density of cattle is negatively correlated with image feature points and positively correlated with image feature noise. Consequently, the recognition effectiveness of beef cattle behavior varies with different levels of cattle density. This paper selects three scenarios of cattle farming—moderate density, high density, and low density—to compare recognition performance with the original YOLOv8n and validate the feasibility of beef cattle behavior recognition using YOLOv8n_BiF_DSC. The comparative results are shown in the following figure.

In the context of beef cattle farming scenarios, the term “low density” refers to images with fewer than six cattle, and the cattle behaviors are generally unobstructed. “Moderate density” corresponds to images featuring a cattle quantity between 6 and 12, with some instances of cattle behavior being partially obstructed. “High density” indicates images with a cattle quantity exceeding 12, where cattle behaviors are significantly obstructed.

From the above figure, when conditions are low-density, both YOLOv8n_BiF_DSC and YOLOv8n accurately identify cattle drinking and standing activities via images. Under moderate-density conditions, YOLOv8n misses one standby event due to the impact of background interference. In high-density scenarios, with increased cattle occlusion and significant background disturbance, YOLOv8n misclassifies two searching events and omits one stand-alone incident. Additionally, YOLOv8n_BiF_DSC demonstrates a higher degree of confidence in its detection bounding boxes. In conclusion, the YOLOv8n_BiF_DSC model effectively generalizes and shows robust performance under differing cattle densities, occlusions, and background interference.

## 4. Discussions

To reduce the difficulty of beef cattle posture recognition and improve accuracy, an increasing number of practitioners are exploring non-wearable recognition based on deep learning models. This approach effectively reduces the manual labor and costs involved in beef cattle posture recognition, providing new possibilities for the welfare, systematization, and intelligence of beef cattle farming. Jingqiu et al. [37] proposed an image-entropy-based target recognition method. They calculated the minimum bounding box and contour mapping for the real-time capture of cow behaviors and hoof and back features, enabling the rapid and accurate identification of breeding and health behaviors in cows from large-scale surveillance videos, thereby enhancing the production efficiency of large-scale farming. Wu et al. [38] presented a method that combines convolutional neural networks and long short-term memory algorithms to identify five basic behaviors of individual cows: drinking, ruminating, walking, standing, and lying. Using VGG16 as the network backbone to extract basic behavioral features from videos and input them into a bidirectional long short-term memory (Bi-LSTM) model for classification, this approach achieves an accurate recognition of basic cow behaviors, providing data support for the physiological health assessment of cows. Zhang et al. [39] proposed a model based on the SlowFast architecture with the addition of the Convolutional Block Attention Module (CBAM) attention mechanism to enhance the model’s spatiotemporal action perception capabilities. The improved model demonstrated a 3.1% enhancement in the recognition of cow behaviors, including standing, lying, walking, drinking, and eating, compared to the base model.

The experiments reported in this paper indicate that the YOLOv8n_BiF_DSC model can accurately identify nine behaviors of beef cattle in various complex scenarios, such as strong light, low light, moderate density, and high density. The recognized behaviors include standing, lying, mounting, fighting, licking, eating, drinking, walking, and searching. The model exhibits a fast recognition efficiency and high accuracy, addressing, to some extent, the challenges of a high difficulty and low accuracy in traditional beef cattle behavior recognition. In the future, by integrating it with online beef cattle behavior detection platforms, practitioners can observe beef cattle round the clock, facilitating the statistical and predictive analyses of crucial aspects such as calf and maternal beef cattle growth. Additionally, it enables the timely detection and management of unexpected issues, such as climbing and fighting among beef cattle.

However, this study has certain limitations due to the relatively homogeneous nature of the beef cattle breed involved. To better serve modern beef cattle farming, the next steps will involve collecting behavior images from a more diverse range of beef cattle breeds and further refining the algorithm’s performance to enhance the accuracy and practicality of beef cattle behavior recognition.

## 5. Conclusions

Traditional beef cattle behavior recognition suffers from difficulties and a low accuracy. In this study, we addressed these challenges by creating a dataset with nine categories of beef cattle behaviors. We propose a non-invasive recognition algorithm called YOLOv8n_BiF_DSC, which combines snake-shaped convolution and attention mechanisms. This approach improves the accuracy of beef cattle behavior recognition. The details are as follows:

(1) In terms of the dataset, this study provides a more extensive range of beef cattle behaviors compared to similar researchers. It includes nine types of beef cattle behaviors: standing, lying, mounting, fighting, licking, eating, drinking, walking, and searching. Additionally, the dataset covers more complex scenarios, such as low lighting, strong lighting, normal lighting, dense populations, sparse populations, and small targets at long distances. This comprehensive dataset closely reflects the actual breeding conditions of beef cattle, providing robust data support for modernized cattle farming and early warning systems.
(2) In terms of algorithms, this study introduces a novel and efficient YOLOv8n_BiF_DSC algorithm for the non-intrusive recognition of beef cattle behavior. The algorithm incorporates the following improvements: the introduction of dynamic snake-form convolution modules to enhance feature extraction capabilities, expand the receptive field, and strengthen model robustness; the integration of the BiFormer attention mechanism to dynamically and sparsely learn critical features, improving the average accuracy of beef cattle posture recognition under typical conditions, as well as addressing challenges like missed detections, false positives, and low confidence in complex scenarios such as dense cattle populations, long distances, and small targets.
(3) In practical scenarios involving beef cattle in low light, strong light, dense groups, and sparse groups, the YOLOv8n_BiF_DSC algorithm achieves a recognition accuracy of 93.6% and an average precision of 96.5% at thresholds of 50, 95, and 71.5%, respectively. This represents improvements of 5.3%, 5.2%, and 7.1% over the original YOLOv8n. The algorithm also outperforms other state-of-the-art algorithms, providing technical support for the intelligent recognition and management of beef cattle behavior.

In the future, efforts will continue to be made to expand the dataset, validate the algorithm’s performance in diverse scenarios, and enhance its robustness. This will involve validating the algorithm’s performance, improving its robustness, and providing technical support for the healthy breeding and systematic management of beef cattle.

## Figures and Tables

**Table 1:** Beef cattle behavior determination rules and example information.

Behavior Number	Behavioral Categories/Labels	Behavioral Description	Instances
(1)	standing	Supported by 4 cow legs, cow head not touching the ground	10,422
(2)	lying	Legs bent and touching the ground or body touching the ground	10,800
(3)	mounting	A cow’s front hooves resting on another cow’s back loin	1518
(4)	fighting	2 or more bulls headbutting each other	5800
(5)	licking	Tongue makes contact in sweeping motion repeatedly with another cow	1600
(6)	eating	Cow’s head enters the trough	6000
(7)	drinking	Cow’s head into the water trough	1680
(8)	walking	4 cow legs displaced from the body, cow’s head not touching the ground	5400
(9)	searching	Cow’s head near or touching the ground	10,300

**Table 2:** Hardware and software configuration information.

Software and Hardware	Parameters
CPU	Intel Core i7-13700F 3.0 GHz
Opencv	4.8.0.76
CUDA	12.1
Cudnn	8700
GPU	NVIDIAGeForce RTX4070(NVIDIA Corporation, Santa Clara, CA, USA)
Operating system	Windows11
Frameworks	Pytorch 2.0.0

**Table 3:** Different convolutional recognition results.

Model	P	R	mAP50	mAP50:95
YOLOv8n	0.883	0.866	0.913	0.644
YOLOv8n_DC	0.917	0.891	0.939	0.671
YOLOv8n_DSC	0.933	0.894	0.945	0.677

**Table 4:** Different attention recognition results.

Model	P	R	mAP50	mAP50:95
YOLOv8n	0.883	0.866	0.913	0.644
YOLOv8n_SE	0.872	0.888	0.91	0.639
YOLOv8n_CBAM	0.911	0.897	0.926	0.656
YOLOv8n_CA	0.915	0.901	0.932	0.664

**Table 5:** Ablation experiment identification results.

Model	DSC	BiF	P	R	mAP50	mAP50:95
YOLOv8n	-	-	0.883	0.866	0.913	0.644
YOLOv8n_DSC	√	-	0.933	0.894	0.945	0.677
YOLOv8n_BiF	-	√	0.922	0.931	0.94	0.672
YOLOv8n_BiF_DSC	√	√	0.936	0.929	0.965	0.715

**Table 6:** Other model identification results.

Model	P	R	mAP50	mAP50:95
FastRCN	0.862	0.843	0.879	0.605
YOLOX	0.869	0.859	0.901	0.639
YOLOv7	0.87	0.862	0.911	0.642
YOLOv8n_BiF_DSC	0.936	0.929	0.965	0.715

## Data Availability

The data presented in this article are available on request from the corresponding author. The data are not publicly available due to privacy and confidentiality.
